# ShockOmics: multiscale approach to the identification of molecular biomarkers in acute heart failure induced by shock

**DOI:** 10.1186/s13049-016-0197-4

**Published:** 2016-01-28

**Authors:** Federico Aletti, Costanza Conti, Manuela Ferrario, Vicent Ribas, Bernardo Bollen Pinto, Antoine Herpain, Emiel Post, Eduardo Romay Medina, Cristina Barlassina, Eliandre de Oliveira, Roberta Pastorelli, Gabriella Tedeschi, Giuseppe Ristagno, Fabio S. Taccone, Geert W. Schmid-Schönbein, Ricard Ferrer, Daniel De Backer, Karim Bendjelid, Giuseppe Baselli

**Affiliations:** Dipartimento di Elettronica, Informazione e Bioingegneria, Politecnico di Milano - Piazza Leonardo da Vinci, 32-20133 Milan, Italy; Custom Software & Electronics SL, Barcelona, Spain; Hemodynamic Research Group, Université de Genève, Geneva, Switzerland; Anesthesiology Department, Hôpitaux Universitaires de Genève, Geneva, Switzerland; Department of Intensive Care, Hôpital Erasme - Université Libre de Bruxelles, Brussels, Belgium; Hospital Universitari Mutua de Terrassa, Terrassa, Spain; Dipartimento di Scienze della Salute, Università degli Studi di Milano, Milan, Italy; Fondazione Filarete, Milan, Italy; Parc Científic de Barcelona, Barcelona, Spain; IRCCS-Istituto di Ricerche Farmacologiche Mario Negri, Milan, Italy; Dipartimento di Scienze Veterinarie e Sanità Pubblica, Università degli Studi di Milano, Milan, Italy; Department of Bioengineering, University of California San Diego, La Jolla, CA USA

**Keywords:** Shock, Acute heart failure, Biomarkers, Transcriptomics, Proteomics, Metabolomics, Hemodynamics, Multiscale modeling

## Abstract

**Background:**

The *ShockOmics* study (ClinicalTrials.gov identifier NCT02141607) is a multicenter prospective observational trial aimed at identifying new biomarkers of acute heart failure in circulatory shock, by means of a multiscale analysis of blood samples and hemodynamic data from subjects with circulatory shock.

**Methods and Design:**

Ninety septic shock and cardiogenic shock patients will be recruited in three intensive care units (ICU) (Hôpital Erasme, Université Libre de Bruxelles, Belgium; Hospital Universitari Mutua Terrassa, Spain; Hôpitaux Universitaires de Genève, Switzerland). Hemodynamic signals will be recorded every day for up to seven days from shock diagnosis (time T0). Clinical data and blood samples will be collected for analysis at: i) T1 < 16 h from T0; ii) T2 = 48 h after T0; iii) T3 = day 7 or before discharge or before discontinuation of therapy in case of fatal outcome; iv) T4 = day 100.

The inclusion criteria are: shock, Sequential Organ Failure Assessment (SOFA) score > 5 and lactate levels ≥ 2 mmol/L. The exclusion criteria are: expected death within 24 h since ICU admission; > 4 units of red blood cells or >1 fresh frozen plasma transfused; active hematological malignancy; metastatic cancer; chronic immunodepression; pre-existing end stage renal disease requiring renal replacement therapy; recent cardiac surgery; Child-Pugh C cirrhosis; terminal illness. Enrollment will be preceded by the signature of the Informed Consent by the patient or his/her relatives and by the physician in charge.

Three non-shock control groups will be included in the study: a) healthy blood donors (*n* = 5); b) septic patients (*n* = 10); c) acute myocardial infarction or patients with prolonged acute arrhythmia (*n* = 10).

The hemodynamic data will be downloaded from the ICU monitors by means of dedicated software. The blood samples will be utilized for transcriptomics, proteomics and metabolomics (“*-omics*”) analyses.

**Discussion:**

*ShockOmics* will provide new insights into the pathophysiological mechanisms underlying shock as well as new biomarkers for the timely diagnosis of cardiac dysfunction in shock and quantitative indices for assisting the therapeutic management of shock patients.

## Background

The clinical protocol “*ShockOmics*: Multiscale Approach to the Identification of Molecular Biomakers in Acute Heart Failure Induced by Shock”, (ClinicalTrials.gov identifier NCT02141607) is part of a complex project which combines the observational clinical study described in this manuscript with animal research and *in vitro* experiments to investigate the fundamental mechanisms of acute heart failure (AHF) in circulatory shock. The goals of the project are the identification of novel biomarkers of shock-induced AHF, the formulation of a multiscale approach to the diagnosis and interpretation of the disease, and the design of new technologies and therapeutic strategies for improving the delivery of care in shock patients.

Circulatory shock is a life-threatening clinical condition, characterized by low tissue perfusion and ensuing cellular damage and organ dysfunction, which affects about one third of patients admitted to the intensive care unit (ICU) [1], with very high mortality rates. Four types of shock are commonly defined according to their hemodynamic patterns: hypovolemic shock (e.g., hemorrhagic shock (HS)), cardiogenic shock (CS), distributive shock (e.g., septic shock (SS)), and obstructive shock [1, 2].

Septic shock is the most common form of shock, which can occur as a complication of sepsis caused by infection. Sepsis and ensuing shock are the leading cause of mortality in the ICU, among the top 15 leading causes of death overall, with a reported mortality rate above 40 %, and more than 200,000 deaths per year in the United States only [3–9]. Recent trials have reported lower mortality rates around 30 % [10–12].

Cardiogenic shock is the second most frequent form of shock, caused by sudden heart failure, due for instance to a severe heart attack. Novel technologies (e.g. extracorporeal membrane oxygenation (ECMO)) have contributed to a reduction of the mortality of cardiogenic shock below 50 % [13–15], but its lethality remains extremely high.

Hemorrhagic shock is a form of hypovolemic shock due to blood loss. It occurs in 36–39 % of the victims of trauma resulting from accidental injuries, which is the leading cause of death at the age between 1 and 44 in the U.S.A. [16, 17].

A frequent consequence of circulatory shock is the development of multiple organ failure (MOF), a condition in which organs not directly affected by the original insult become dysfunctional and eventually contribute to poor outcome. In the specific case of the heart, its function can be directly impaired by cardiac disease, such as acute myocardial infarction or prolonged arrhythmias, which causes cardiogenic shock and low cardiac output. However, in the other forms of shock, the heart can fail in the general context of MOF [18, 19]. Although the goal of restoring an adequate level of perfusion is to prevent any organ from failing, the protection of the heart and the preservation of its function are instrumental for ensuring hemodynamic stability, and an adequate perfusion of all vital organs.

The relationship between shock, hypotension and hemodynamic instability, inflammation and MOF has been extensively investigated, but the mechanisms that ultimately trigger molecular and cellular injury which cause tissue and organ dysfunction remain largely undetermined, so that there is no clear therapeutic target. Hence, current therapies are targeted to restoration of hemodynamic variables and reduction of symptoms of shock/MOF, but they are unable to act at the “beginning of the cascade”.

Vital signs available from measurements or estimates in critical care settings, such as electrocardiogram (ECG), arterial blood pressure (ABP), central venous pressure (CVP), stroke volume (SV) and cardiac output (CO), pulmonary artery pressure (PAP), pulmonary capillary occluded pressure (PAOP), and photo-plethysmographic (PPG) pulse-oximetry are routinely used for monitoring purposes. They convey instantaneous information on the CV status of the patient, but they cannot accurately predict the occurrence of hypotensive episodes and are often not sufficient to guide a timely and effective Early Goal Directed Therapy [20–22], because of the poor understanding of the pathological cascades characterizing shock [23–34]. As a consequence, the success of any intervention, such as fluid resuscitation, vasopressor therapy and the use of inotropes is limited and depends on the trade-off between their short-term beneficial effect and their potential long-term risk. Fluids may restore blood pressure within minutes, but serious complications such as pulmonary edema may arise thereafter as a consequence of excessive fluid administration, causing alterations in tissue perfusion, delay in organ recovery and prolonged supportive therapies, such as mechanical ventilation. Vasoactive drugs are used to maintain blood pressure but present the risk of increasing the cardiac afterload and/or facilitating the occurrence of arrhythmias. Hence, their use can further stress the heart, potentially hampering its residual functionality [32–34].

In the context of low blood pressure, low flow, and reduced ventricular contractility typical of circulatory shock and hemodynamic instability, careful monitoring of heart function is essential to prevent further tissue hypoperfusion and subsequent organ failure. *ShockOmics* focuses on acute heart failure due to shock, and will explore the presence of biomarkers, which could be related (but not limited) to a cascade of pathogenic phenomena, whose onset is potentially associated to the role of the intestine and of the endocrine glands as a source of powerful mediators for tissue injury [26, 35]. *ShockOmics* aims at investigating the molecular triggers of AHF associated with shock and to identify inflammatory mediators and markers which are activated after an initial insult, with a particular emphasis on the role of uncontrolled proteolytic activity as a major cause of severe tissue injury. To achieve this goal, a systematic analysis of expression levels of transcripts, genes and their protein products, and of peptides generated by proteolysis will be carried out on blood samples obtained from ICU patients hospitalized. Based on the preliminary identification of candidate biomarkers of shock-induced AHF, animal experiments and in vitro studies will further validate the initial hypotheses on the fundamental mechanisms and on the biomarkers of the disease. This approach, besides focusing on new biomarkers of the disease, will aim to define new targets for therapy, in order to overcome the shortcomings of current therapies for circulatory shock. The final outcome of *ShockOmics* will be a multiscale integration of the information from different scales of investigation, from gene expression, to protein synthesis and metabolite expression, to organ specific injury in the heart and hemodynamic patterns characterizing the alteration of CV function in shock. In this framework, an important aspect is the interpretation of  the patho-physiological changes in system level variables, i.e. CV measurements, which are routinely available in emergency departments, ICUs and operating rooms, in function of the fundamental processes of disease.

The objectives of the clinical study included in the framework of the *ShockOmics* project will specifically address the following questions:

- What are the circulating biomarkers of AHF and subsequent hemodynamic instability?
- What is the relationship between such biomarkers, the biochemical parameters, and the hemodynamic measurements, which are routinely available in the ICU?
- What are the regulatory points in the cascade, which could become targets for a cardioprotective therapy against shock?

The expected outcomes of the clinical study will be:

1. Definition of candidate biomarkers by means of cutting edge –omics techniques;
2. Development of an innovative multiscale, systems biology based models to describe the relationship between hemodynamic measurements/waveforms available in ICU/OR/ED and the progression of shock induced AHF;
3. Identification of novel targets for effective cardioprotective therapies to prevent/contrast shock progression;
4. Creation of a complex and detailed database of hemodynamic signals collected from the ICU monitors.

## Methods and design

The *ShockOmics* clinical protocol defines the specifics of a multicenter prospective observational study meant to evaluate molecular mechanisms underlying shock and associated with AHF.

The ICUs involved in the clinical study are located in the following hospitals:

- Hôpitaux Universitaires de Genève, Université de Genève (Geneva, Switzerland)
- Hôpital Erasme, Université Libre de Bruxelles (Brussels, Belgium)
- Hospital Mutua de Terrassa (Terrassa, Spain)

All Institutional Ethical Committees in the three participating institutions approved the clinical protocol.

*ShockOmics* will recruit severe SS and CS patients. Severe HS patients will be also recruited, but their inclusion in the study will be limited to the system level investigation of the features of the hemodynamic signals, while the –omics analyses will not be carried out for this group (see Discussion section).

The study will recruit 90 patients suited for analysis with full data collection available, i.e., well preserved blood samples, high quality hemodynamic recordings, all clinical, anamnestic, diagnostic and prognostic data. The patients will be classified according to existing shock severity scores, such as the Sequential Organ Failure Assessment (SOFA) score and the Acute Physiology And Chronic Health Evaluation (APACHE) II [36]. In particular, the SOFA score will be utilized to set the threshold of shock severity as one of the inclusion criteria of the study (see *Inclusion and Exclusion Criteria*).

### Sample size

The sample size was computed taking into consideration the following assumptions:

1. *ShockOmics* is an exploratory observational study aimed at testing new hypotheses on the molecular mechanisms of shock and consequent secondary organ failure, particularly AHF. Such hypotheses will be further explored and validated in animal models.
2. Incidence of AHF in the population is low (average estimate 1.3 cases/1,000 subjects, increasing with age up to 11.6/1,000 in those aged 85 years and over) [37, 38]. Conversely, MOF and specifically AHF are a frequent and often lethal complication in shock patients. Shock increases re-hospitalization rates, organ failure and associated mortality even after ICU discharge [39]. Therefore, we expect to observe the activation of inflammatory factors and pathogens because of the local and systemic ischemia/reperfusion episodes occurring in shock, with an increased risk of AHF.
3. Based on experimental and preclinical studies, we expect to test the occurrence of uncontrolled proteolytic activity, caused by serine proteases, metalloproteinases and other enzymes.

The sample size of *n* = 90 patients was calculated considering as outcome a quantitative measurement of cardiac function (i.e. cardiac output), and the following parameters: a) an Effect Size (f^2^) of 0.205 (for a strength of association equal to R^2^ 0.17); b) α type I error probability equal to 0.05; c) power = 81 %, using a multiple linear regression; d) 10 predictors tested in the model and giving the effect size.

If the strength of association (R^2^) for the selected set of predictors is equal to 0.20, the Effect Size (f^2^) would increase to 0.25, giving to the study a Power of 89 %, while for models with R2 higher than 0.24 the Power obtained would be higher than 95 %.

The analyses of the blood samples and hemodynamic measurements will entail both analyses of the total sample (*n* = 90), which will allow the identification of common predictors across different types of shock, and of the subgroups of the different types of shock, which will allow to identify specificities associated to the disease etiology. A minimum sample size of *n* = 30 patients is needed per each subgroup in order to identify a set of predictors producing an Effect Size equal to 0.43 (for a strength of association equal to R^2^ 0.30), testing up to 3 predictors, with a Power of 80 %, and type I error α equal to 0.05. A maximum sample size of *n* = 60 patients will enable to identify a set of predictors producing an Effect Size equal to 0.25 (for a strength of association equal to R^2^ 0.20), testing up to 6 predictors with a Power of 80 %, and type I error α equal to 0.05.

In the specific case of hemorrhagic shock not determined by pre-existing pathologies (e.g., trauma patients), we expect the risk factors of low perfusion (i.e., reduced cardiac output) in these patients to be strictly related to shock. HS patients will be considered as reference shock population.

We will use data from septic patients and patients with acute heart disease not in shock together with septic shock and cardiogenic shock patients to conduct logistic regression analysis. Each subgroup analysis will be run on minimum of 40 subjects, allowing to identify single predictors with an Odds Ratio of 2.85 on a binary response variable defined as “shock onset”, with a Power of 83 % and type I error α equal to 0.05.

The sample size was estimated using G*Power 3 Calculator. We estimate that each ICU will recruit *n* = 50 patients in order to reach the above mentioned sample size, considering a drop out rate of ~40 %.

### Patient groups

#### *Septic shock* (*n* = 60)

Septic shock is defined as sepsis-induced hypotension, characterized by systolic blood pressure < 90 mmHg or a drop of > 40 mmHg from baseline or mean arterial pressure < 65 mmHg, persisting despite adequate fluid resuscitation. Adequate resuscitation will be considered achieved when a volume of at least 30 ml/kg of crystalloids will be administered. Additional fluid resuscitation will be guided using hemodynamic monitoring according to local practices. Subgroup analyses will be undertaken to compare the community-acquired and nosocomial-acquired infections.

#### *Cardiogenic shock* (*n* = 30)

Cardiogenic shock is defined as a state of inadequate circulation of blood because of ventricular failure due to acute cardiac conditions, such as acute myocardial infarction (AMI) or prolonged arrhythmias (PA). Additionally, characteristics of the CS patients recruited in *ShockOmics* will be: hypotension, characterized by systolic blood pressure < 90 mmHg or a drop of > 40 mmHg from baseline or mean arterial pressure < 70 mmHg, persisting despite adequate fluid resuscitation; Cardiac Index <1.8 L/min/m^2^ without supports or < 2.0–2.2 L/min/m^2^ with the support of inotropic drugs (e.g. dobutamine/isoprenaline/phosphodiesterase inhibitors or levosimendan) or of cardiac assistance devices; cardiac overload or altered left/right ventricular function are assessed by typical echocardiographic indices [15]:

- Left ventricular (LV) volume and ejection fraction (EF) estimated by the apical biplane method of disks (modified Simpson’s rule):
  - EF <35 % = systolic heart failure
  - LV diastolic volume/ body surface area (BSA) > 97 ml/m2 = severe LV dilation
- Aortic velocity time integral (VTI) measured by PW Doppler on LVOT:
  - Abnormal if < 18 cm
  - Severely abnormal if < 14 cm
- Mitral annular diastolic PW tissular Doppler velocities:
  - Lateral E/E’ >12 = high LV filing pressure
  - E/E’ <8 = normal LV filing pressure
- Right ventricular /left ventricular (RV/LV) diameter (or cross section area) ratio > 1 = severe RV overload (intermediate if between 0,6 and 1).
- Paradoxical inter-ventricular septum motion (or “D-shape”) = severe RV overload

#### Hemorrhagic shock (*n* = 15)

Hemorrhagic shock is a hypovolemic form of shock, characterized by the rapid loss of significant amounts of blood, systolic blood pressure < 90 mmHg or a drop of > 40 mmHg from baseline or mean arterial pressure < 65 mmHg. The HS patients enrolled in the study will be analyzed exclusively from the standpoint of their hemodynamic records and clinical data.

#### Control groups (*n* = 30)

The *ShockOmics* study will also entail the collection of blood samples and hemodynamic data when possible in comparison groups of healthy subjects or patients not it shock:

- *n* = 5 healthy blood donors will be collected for the purposes of obtaining reference values for proteomics analysis
- *n* = 10 septic patients not in shock: sepsis caused by infections with inflammatory response with one organ dysfunction at most (as indicated by SOFA sub-scores for specific organ function), without organ support therapy (i.e., mechanical ventilation, renal replacement therapy) and with lactate levels < 2 mmol/L
- *n* = 10 cardiac patient not in shock, affected by AMI or PA patients

### Inclusion and exclusion criteria

The inclusion criteria of *ShockOmics* are:

- SOFA score > 5
- First blood sample and first hemodynamic measurements available within 16 h from admission to the ICU
- Informed Consent available: the consent will be requested to the patient, or to its relatives in case of altered consciousness, and signed by the physicians responsible for *ShockOmics* in the ICU. Delayed consent may be asked according to local rules and regulations in case the relatives were unavailable at the time of potential enrollment

The exclusion criteria of *ShockOmics* are:

- Risk of fatal illness and death within 24 h
- Patients already enrolled in other interventional studies
- More than 4 units of red blood cells transfused
- Patients receiving plasma or whole blood
- Active hematological malignancy
- Metastatic and/or active cancer
- Immunodepression, including transplant patients; patients infected by the human immunodeficiency virus (HIV+); constitutive immune system deficiency; any immunosuppressive therapy, including oral and parenteral corticosteroids (aerosols are allowed)
- Patients with pre-existing end stage renal disease needing renal replacement therapy. The introduction of continuous veno-venous hemofiltration from the day of admission onward is allowed
- Cardiac surgery in the previous ten days
- Child-Pugh C cirrhosis or acute liver failure
- Terminal illness

### Timeline of data collection

- Time 0 (T0): admission to the ICU with diagnosis of shock or time of shock diagnosis in patients initially admitted without shock symptoms (see inclusion/exclusion criteria above);
- Time 1 (T1): time at which the first blood sample for analysis is collected, within 16 h after T0. This time point is considered representative of acute shock before the therapy has taken effect, when shock has already activated the main patho-physiological cascades of inflammation and disease;
- Time 2 (T2): time at which the first blood sample for analysis is collected, at 48 h after T0. At this time point, the treatment has been administered for a long enough amount of time to evaluate its effects on the early molecular markers of disease;
- Time 3 (T3): time at which the third blood sample for analysis is collected, on day 7 of the ICU stay of the patient or before discharge from the ICU in case of shorter stays or before discontinuing therapy (death). At this time point, the activated molecular pathways and the relevant system level consequences are assumed to have reached a steady state condition;
- Time 4 (T4): follow up (FU) on ~ day 100 from Time 0. A blood sample will be collected in survivors who will not be re-hospitalized for the consequences of shock during the time between T3 and T4.

**Fig. 1:**
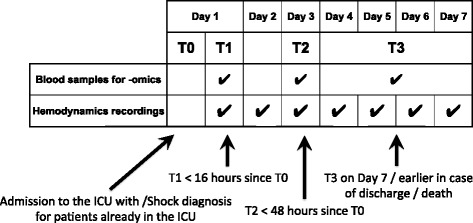
Time course of the monitoring of a patient enrolled in the *ShockOmics* study (hemodynamic data acquisition and blood samples). The hemodynamic data are collected daily during the observation window (up from time 0 on day 1 up to day 7). The total number of blood samples is 3: the first sample is collected at T1, the second at T2, and the third at T3, which can occur on any day between day 4 and day 7 (T3 occurring on day 7 in case of ICU stay longer than 7 days or on day 4–6 in case of early discharge or death)

The time course of data collection for the protocol is shown in Fig. 1.

### Clinical endpoints

The clinical endpoints that will be used to evaluate shock progression are:

- AHF assessed by a pool of measurements/estimates of cardiac function, including cardiac output, filling pressures, inotropic drugs requirements, left and right ventricles function estimated by echocardiography. AHF will be at evaluated at Time 1, 2, 3
- Mechanical ventilation free days or organ support -days
- ICU survival
- Outcome at discharge from the ICU (objective: dead or alive, morbidities; subjective)
- Outcome at discharge from the hospital (objective: dead or alive, morbidities; subjective)

### Clinical data collection

The following anamnestic and clinical data will be recorded daily from T1 until T3:

- Laboratory data from urine and blood samples
- Comorbidities
- Severity scores (SOFA and APACHE II scores)
- Time from onset of symptoms to shock diagnosis
- Time from ICU/ER admission to shock diagnosis
- Biochemical parameters, e.g. lactate levels, pH, etc.
- Therapy (drugs, fluids, etc.), temperature, urine output, stool production, bowel sound, abdominal fullness, appetite, food and fluid consumption or fasting, nausea and vomiting
- Cardiorespiratory assistance (mechanical ventilation, ECMO, intra-aortic balloon pump, etc.)
- Assessment of organ failure, and in particular heart failure
- Cognitive assessment (encephalopathy) - by SAS, RASS and CAM-ICU scale
- For the patients not discharged from the ICU on day 7, the clinical data will be recorded on day: 10/15/22/30 and every 15 days until discharge from the ICU

### Biological samples

Urine and blood samples will be collected at the times T1, T2, and T3. The typical management of shock patients in ICU requires arterial, venous and urinary catheterization. These accesses are routinely used for drug/fluid administration and will also be used for the collection of the samples for analysis.

The ICU staff will contact survivors in order to schedule a follow-up visit on day 100 (T4, Recovery), during which a blood sample will be withdrawn for analysis.

The collection and analysis of urine will permit to evaluate standard biochemical parameters, routinely required to integrate the information from blood analysis, such as for instance kidney function, liver function, medullary function, etc.

### Hemodynamic monitoring

The hemodynamic data will be downloaded from the bedside ICU monitor on a laptop computer by means of dedicated software. Details on the implementation of the solutions to the technical issues of downloading signals from the monitors and on the acquisition protocol are addressed in the Discussion section.

The continuous waveforms, which will be routinely accessible, include:

- ECG
- ABP, via an arterial catheter inserted in the femoral or radial artery
- CVP, in patients with a central venous line with a pressure transducer
- PPG
- Respiratory signals: bio-impedance or thoracic belt
- Continuous SpO2

Further, continuous and non-continuous measurements and estimates, which will be available intermittently, will include:

- Continuous measurements of PAP, PAOP, and estimates of CO via thermodilution, by means of a pulmonary artery catheter, when in use
- Stroke volume, CO, global end diastolic volume, extravascular lung water from trans-pulmonary thermodilution (PiCCO ® [Pulsion Medical System]) when in use
- Trans-thoracic or trans-oesophageal echocardiographic assessment on admission, on Day1, Day2, Day3, Day7/Discharge/Death
- Ventilation parameters (settings of mechanical ventilation if applicable; including pressure, tidal volume, static compliance, etc.) on Day1, Day2, Day3, Day7/Discharge/Death
- Central or mixed venous oxygen saturation and artero-venous pCO_2_ difference on Day1, Day3, Day7/Discharge/Death.

### Protocol documentation

Each Experimental Center will archive the patient documentation in a Trial Centre File, which will include:

- Study Protocol signed by all relevant individuals
- European Commission Approval Notification
- Administrative Approval Notification (from the hospital)
- Any relevant correspondence/contracts between Funding agency (EU) and the *ShockOmics* consortium
- Completed transcoding file: including the transcoding from anonymized study codes and patient name, last name, date of birth, etc.
- Informed Consent and Privacy
- Individual data of the patient, i.e. Case Report Form (CRF) and hemodynamic tracings, dated and signed by the Investigator in charge for the center
- Safety Session: including all documentation related to severe adverse events incurred during the hospitalization

## Discussion

In this section, we discuss the solutions that were devised to address the main technical issues faced during the set-up stage of the clinical protocol, such as: high fidelity hemodynamic waveform download from the bedside monitor and storage in a database; guidelines for the treatment of blood samples to be used for –omics analyses; the definition of control groups; the inclusion in the study of a hemorrhagic shock group and the relevant limitations; the timeline of the project.

### Software for the download of the hemodynamic signals from the monitors, CRF and database

The three ICUs participating in *ShockOmics* are equipped with different bedside monitors:

- Hôpitaux Universitaires de Genève: Philips Intellivue MP70 ® (version K)
- Hôpital Erasme, Université Libre de Bruxelles: Dräger INFINITY (C700 + M540 ®)
- Hospital Mutua de Terrassa: Philips Intellivue MP70 ® (2012 Software)

A laptop computer will be connected to the monitor and hemodynamic and ventilation signals will be synchronously downloaded by means of dedicated software programs.

The following software will be used:

- Hôpitaux Universitaires de Genève: ixTrend Professional 2.0 ® (ixellence GmbH, Wildau, Germany)
- Hôpital Erasme, Université Libre de Bruxelles: NOTOCORD-hem Evolution ® (NOTOCORD Systems SAS, France)
- Hospital Mutua de Terrassa: Better Care software ® (Better Care S.L., Sabadell, Spain).

The signals, which will be typically downloaded from the monitors and stored in the database, include:

- Electrocardiogram at a sampling frequency, fs = 500Hz, 200Hz, 1000Hz
- Central arterial blood pressure, fs = 125 Hz, 200Hz
- Central venous pressure when a central venous line is present, fs = 200 Hz
- Pulse oximeter photoplethysmography signals, fs = 200Hz, 125Hz
- Respiratory signals: bioimpedance, fs = 62.5 Hz; thoracic belt, fs = 100Hz, 200Hz.

The different sampling rates are due to the different monitors and acquisition systems.

Prior to the implementation of the software-monitor interface, pilot tests on signals collected by the different software programs were run to verify the reliability of the acquisition procedures and the quality of the signals for the computation of the derived variables (e.g. systolic blood pressure series) which are required for the subsequent mathematical analysis. The exportation of data in the same format (e.g., as .csv files) ensures that there is no difference in the treatment of the signals by the software that could potentially hamper the consistency of the analysis, independent of the interface implemented to record the data, and also of the ICU monitor.

The signals will be uploaded in the patient CRF, which is stored in the *ShockOmics* database. The CRF and the database architecture were developed by the *ShockOmics* partner Custom Software & Electronics, SL, Barcelona, Spain. The CRF is a multi-field electronic file in which the clinical data and hemodynamic signals collected during the ICU stay of a patient enrolled in the protocol are stored.

An appropriate signal collection protocol was designed, in order to generate a uniform hemodynamic database, and to optimize the workload of the physicians in the ICUs. Typical recordings will take place after routine patient washing. ECG, hemodynamic, and respiratory signals will be downloaded to the laptop and real time annotations will be carefully taken during standard maneuvers to test the hemodynamic status of the patients, such as end expiratory occlusion test in deeply sedated patients, fluid challenge, vasopressor challenge, etc. Besides the inclusion of specific maneuvers in the hemodynamic recordings, longer recordings without the presence of specific maneuvers may be collected in order to evaluate the long-term dynamics in the cardiovascular signals.

The hemodynamic signals will be analyzed by means of advanced mathematical techniques in order to derive clinically relevant information from the available waveforms (e.g. cardiac output estimates, stroke volume and pulse pressure variation in correspondence of fluid challenges, cardiovascular variability indices such as baroreflex sensitivity, non linear indices of heart rate and blood pressure dynamics, etc.).

The recordings will be collected every day during the window of observation defined in the protocol (see above, 7 days in case of early discharge from the ICU or death).

The database of *ShockOmics* will include all the CRFs of the patients enrolled in the study and all the cardiovascular signals downloaded from the ICU monitors. Further, an appropriate storage space has been foreseen for the most important parameters of the –omics analyses, which will be performed on the blood samples withdrawn from the patients at T1, T2, T3, and T4 when possible. The database will be partially available to researchers who will apply to access it for analysis of the data, following the expression of interest in a collaboration with the *ShockOmics* consortium and a request of access to the database which will be evaluated internally by *ShockOmics* consortium. Partial access to the database (e.g., the hemodynamic recordings) will be made freely available to the scientific community upon completion of the clinical study and of the analyses to be performed by the *ShockOmics* consortium.

### Blood sample handling and pre-processing for –omics analysis

Each of the blood samples (at T1, T2, T3, T4 for the survivors) collected for the –omics analysis will be studied by means of transcriptomics, proteomics, and metabolomics analyses.

A volume equal to 10 mL of blood will be withdrawn at the 4 points in time indicated above, according to the following protocol:

- 1 mL of peripheral venous blood will be withdrawn in Ethylen Diamine Tetra-Acetate (EDTA)
- Subsequently, two aliquots of 0,5 mL of blood will be distributed in two vials containing 0,5 mL of stabilizing reagent, then gently mixed and stored at -20 °C
- Plasma (for transcriptomic studies): 6 mL of blood in EDTA-coated tube, centrifuged at 1300 g for 10 min at 10 °C; the supernatant (plasma ~3 mL) is split into two vials (1.5 mL each) and further centrifuged at 16,000 g for 10 min at 10 °C. Finally, plasma is transferred into two 1.5 mL tubes and stored at -80 °C
- Plasma (for proteomics and metabolomics studies): a 50 % solution of 3 mL of blood in protease inhibitor is transferred in an EDTA-coated tube, centrifuged at 1300 g for 10 min at 10 °C; the supernatant (plasma ~1.5 mL) is split into three vials (0.5 mL each) and stored at -80 °C.

### Control groups

The criteria for the collection of the blood samples in the patients will also be applied to the comparison groups of septic patients and AMI or arrhythmic patients not in shock. Regarding the blood donors, only one sample will be taken and utilized as a healthy control benchmark for the samples from the patients enrolled in the study.

### Hemorrhagic shock

A critical element of the clinical protocol is the inclusion of HS patients in the study. *ShockOmics* is aimed at investigating septic, cardiogenic and hemorrhagic shock in human patients, and in swine shock models. However, the –omics analyses impose extremely restrictive constraints on transfusions of blood and plasma, due to the impossibility of disentangling the confounding factors introduced by the donor blood. For these reasons, it has not been deemed possible to perform the multiscale investigation described for CS and SS in the case of HS patients. The same multiscale approach, aimed at combining –omics experiments with the mathematical analysis of hemodynamic recordings, will be followed in all the three (hemorrhagic, cardiogenic, septic) swine models of shock.

Still, for the sake of consistency with the general framework of the project, a limited number of severe hemorrhagic shock patients will be included in the database, but only the hemodynamic signals will be collected in these patients, without any blood samples and related analysis.

## Trial status

At the time of publication, 64 patients out of the planned 90 have been recruited, and 12 controls out of 20. The study is expected to be completed by the first quarter of 2016.

## Abbreviations

ABP: arterial blood pressure
AHF: acute heart failure
APACHE: acute physiology and chronic health evaluation
CO: cardiac output
CS: cardiogenic shock
CRF: case report form
CVP: Central venous pressure
ECG: electrocardiogram
ECMO: extracorporeal membrane oxygenation
EDTA: ethylen diamine tetra-acetate
EF: ejection fraction
HS: hemorrhagic shock
ICU: intensive care unit
LV: left ventricular
MOF: multiple organ failure
PAP: pulmonary artery pressure
PAOP: pulmonary capillary occluded pressure
PI: protease inhibitor
PPG: photo-plethysmography
RV: right ventricular
SOFA: sequential organ failure assessment
SS: septic shock
